# Risk perception and determinants in small‐ and medium‐sized agri‐food enterprises amidst the COVID‐19 pandemic: Evidence from Egypt

**DOI:** 10.1002/agr.21676

**Published:** 2020-12-03

**Authors:** Assem Abu Hatab, Carl‐Johan Lagerkvist, Abourehab Esmat

**Affiliations:** ^1^ Department of Economics Swedish University of Agricultural Sciences Uppsala Sweden; ^2^ Department of Economics and Rural Development Arish University Arish Egypt; ^3^ Department of Agricultural Economics Al‐Azhar University Assiut Egypt

**Keywords:** COVID‐19, Egypt, probabilistic conditional binary recursive inference algorithm, risk perception, small‐ and medium‐sized enterprises

## Abstract

The coronavirus disease‐2019 (COVID‐19) pandemic has disrupted many activities along agri‐food supply chains in developing countries and posed unprecedented challenges in particular to small and medium agri‐food enterprises (SMEs). Drawing on a survey of 166 Egyptian agri‐food SMEs, this study investigates differences in‐ and determinants of COVID‐19 business risk perception among these enterprises. The empirical results showed that risk perception was highly asymmetric across geographical regions. Enterprises with longer cash flow coverage periods and higher values of total assets perceived significantly lower risk levels, as cash and assets functioned as a buffer against the impact of COVID‐19. The findings of the study imply that the “just‐in‐time” approach and the absence of a proactive and preventative stance to risk management reduced the resilience of agri‐food SMEs to the risks presented by the pandemic. Generally, enterprises that operate both in domestic and export markets perceived lower COVID‐19 risks. Finally, the main export destination to which the surveyed enterprises export was a significant determinant of their risk perception. These findings could be useful to managers of agri‐food businesses in terms of better understanding of risks and promotion of risk management practices. More so, they can help design effective policy interventions to mitigate the impacts of the pandemic on Egyptian agri‐food SMEs and build up their resilience to future pandemics and shocks.

## 1 INTRODUCTION

The coronavirus disease‐2019 (COVID‐19) pandemic has resulted in unprecedented stresses on food supply chains, creating profound challenges for farm labor and production, processing, transport, and logistics as well as major shifts in demand and consumption (OECD, 2020a). Most of these challenges resulted from policies (e.g., nation‐wide lockdowns and restrictions on people's mobility) that were adopted by governments to contain the spread of the pandemic (Fei et al., 2020). These measures have drastically affected the essential flow of food from producers to consumers and put fresh food production and the supply chain under the risk of disruption (Hobbs, 2020). While the majority of countries across the globe have been affected by the spread of COVID‐19, the repercussions of the pandemic on food supply chains in developing countries threaten to further affect the livelihoods of the poor, a majority of whom depend on agriculture, and are expected to extend to food security and stability (Barrett, 2020). In this respect, Jribi et al. (2020) argue that COVID‐19 may have substantial implications for the achievement of the Sustainable Development Goals (SDGs) in developing countries, in particular SDG 2 (End Hunger) and SDG 12 (Ensure sustainable consumption and production patterns).

Downstream stages of the food supply chain in developing countries have been hit harder than upstream stages by the effects of the pandemic (IMF, 2020). This is because the postproduction stages of the food chain take place in periurban and urban areas, where the population density is traditionally greater and the government containment measures are stricter. For a number of reasons, small‐ and medium‐sized agri‐food enterprises (agri‐food SMEs), which represent a fundamental component of the economic fabric of developing countries and account for more than 90% of business and up to 60% of employment (Aga et al., 2015), have been particularly affected by the pandemic. First, agri‐food SMEs in developing countries rely much more heavily on labor than machinery for their activities, and thus the continuity of their businesses has been especially compromised by lockdowns (Lu et al., 2020). Second, the relatively low logistical and financial capacity of these enterprises to implement hygiene and health measures increase their relative vulnerability and many of them face difficulties restarting operations due to binding operational and capital constraints. Third, the fact that food production and distribution in developing countries rely on a large number of agri‐food SMEs, together with the fact that many businesses are informal activities, results in the exclusion of these businesses from stimulus plans that governments offer to larger enterprises (Jola‐Sanchez, 2020).

The impact of COVID‐19 on food supply chains has prompted increasing reflection and attention on the global food system and attracted the attention of both scholars and practitioners (e.g., FAO, 2020; Zurayk, 2020). A closer look at this emerging literature on COVID‐19 and food chains reveals three main characteristics/shortcomings. First, the bulk of the literature is focused on developed countries (e.g., Garnett et al., 2020; Hobbs, 2020; Ker, 2020), whereas there is still a lack of research based on the experiences of developing countries. Second, the literature has so far focused on the very end of the food chains by investigating consumers' purchasing behavior during the pandemic (e.g., Grashuis et al., 2020), food waste (e.g., Aldaco et al., 2020), and food security and nutrition outcomes (e.g., Naja & Hamadeh, 2020). Other studies have focused on the macroeconomic impacts of the pandemic and the responses and mitigation strategies at the policy level (e.g., Arouna et al., 2020). However, there is predominantly anecdotal evidence on the impact of the pandemic on individual stages and actors of the food supply chain, including agri‐food SMEs. Third, the literature is dominated by qualitative and descriptive studies, which is an expected characteristic of an emerging body of literature. That is, existing studies on SMEs in developing countries tend to explore and describe the dynamics of COVID‐19 and its effects on food chains in different contexts, rather than measuring the extent of these effects. In summary, the literature provides ample evidence as to how COVID‐19 presents uncertainties with regard to the business performance of agri‐food SMEs in developing countries, how these enterprises perceive the risks from the pandemic and what factors determine their risk perception, and what risk management strategies are adopted to cope with these uncertainties and impacts. While the overall expectation is that pandemics such as COVID‐19 will occur more often in future, it is particularly important to understand the current impacts of the pandemic on SMEs and their mitigation and adaptation strategies to build resilience to future pandemics (McKee & Stuckler, 2020).

The remainder of this paper is structured as follows. Section 2 provides an overview of the agri‐food SMEs sector in Egypt in the context of the COVID‐19 pandemic. Section 3 discusses the survey and methods used to measure perceived COVID‐19 risks and their determinants. Section 4 reports the empirical results from a survey of 166 agri‐food SMEs (with less than 100 employees) officially registered in Egypt and licensed to operate both in the domestic and international market for agri‐food commodities. Section 5 discusses the main findings and their policy and agribusiness implications.

## 2 AGRI‐FOOD SMES IN EGYPT AND THE PRESENT STUDY

In Egypt, SMEs represent more than 90% of all businesses, almost 60% of the employment, and around 75% of the national value added (AfDB, 2016). Agri‐food SMEs play a vital role in the food and agricultural economy of Egypt: they represent at least 90% of Egyptian agri‐food production and export firms, generate more than 90% of employment, and account for more than 75% of agri‐food exports (MALR, 2015). Despite their vital role, Egyptian agri‐food SMEs are generally more vulnerable than large‐sized enterprises to extreme events and external shocks due to several reasons, including size, lower productivity, few financing options, less diversification in business activities, and weaker financial structure (Abu Hatab & Hess, 2013). Therefore, primary reports indicate that the COVID‐19 outbreak and the subsequent containment measures that the government of Egypt implemented have drastically affected the essential flow of commodities produced by agri‐food SMEs from farms and producers to consumers. With the first confirmed case on 14 February, Egypt is one of the world's COVID‐19 hotspots and one of the most affected countries on the African continent (Shabir & Aijaz, 2020). Between mid‐February and mid‐July, the number of confirmed cases has increased rapidly reaching 82,000 cases, resulting in around 4,000 deaths and making Egypt the second most affected country in Africa, after only South Africa, both in terms of the number of cases and deaths. (WHO, 2020). Many agri‐food SMEs are struggling to cope with unprecedented challenges, including labor shortages, disruptions in supply chains because of transportation problems and other issues, disruptions in the functioning of agricultural markets, and price volatility and changes in consumer purchasing behavior. After governments granted permission to reopen, many SMEs faced difficulties resuming operations, which resulted in further significant economic losses and put many in danger of closing permanently. In this regard, a recent survey of the impacts of the pandemic on 283 Egyptian SMEs shows that 3% of the sample enterprises permanently ceased business activities and 54% stopped temporarily due to confirmed COVID‐19 cases within the enterprises, government instructions, or lack of demand (CHF MCSE, 2020). The results of the same survey revealed that 60% of the participating SMEs faced a shortage of workers, and that the SMEs project an increase in operating costs of between 24% and 50% and a decrease in revenue between 25% and 39%.

It is important to note that the pandemic is still in its infancy. By their very nature, extreme events, such as the COVID‐19 pandemic, may expose SMEs to risks that can have an impact not only their business activities but also ultimately their survival on an organizational level (Sullivan‐Taylor & Wilson, 2009). From a management perspective within an agri‐food SME, a dynamic adjustment process in response to managerial probabilistic‐consequentialist considerations (henceforth: subjective risk assessment) across key business dimensions is going to be a primary focus for a nonnegligible timespan. In the short‐term, risks and adjustments are going to relate to shocks of input (e.g., labor) as well as output markets (e.g., drop in demand and market access challenges). Over the medium‐term, risks and adjustment effects will have impacts both on the input and on the output side. Given this situation, for the design and implementation of effective policy interventions to mitigate the direct impacts on these enterprises, it is important to investigate differences in managerial perceptions of key Covid‐19 related business risks. Furthermore, it is also relevant to examine whether there are systematic drivers of such differences. This forward‐looking perspective, based on perceived risks, is very relevant to agri‐food SMEs since planning is necessary and “bad” decisions can be very costly.

The present study contributes to this specific literature in several important ways. First, it provides fresh empirical evidence on the perception and determinants of risks associated with the COVID‐19 pandemic in relation to agri‐food SMEs in a developing country. Risks perceived by SMEs are generally far different from those encountered by large‐sized enterprises (Thakkar et al., 2009). Likewise, risk perceptions, determinants, and impacts on SMEs may vary considerably between developing and developed countries. Therefore, the analysis in this study can provide useful insights that can help managers and policymakers understand the impacts of the pandemic on this particularly important enterprise segment and develop active risk management strategies, which can in turn lead to improved performance. As illustrated by Koh and Tan (2006), risk identification represents the first step in formulating effective supply chain strategies. The second contribution of this study, in contrast to approaches commonly adopted in the literature that focus on uncertainty and risk avoidance, is the focus on risks through the investigation of the impact risk has on agri‐food SMEs. As shown by Parnell et al. (2015), overreliance on uncertainty avoidance contributed to performance problems in many SMEs. According to Sopha et al. (2020), an examination of uncertainty and its connection to the performance of SMEs in developing countries is still underexamined; however, identifying the sources of uncertainty and their determinants is important as this can enable SMEs to recognize the root causes of issues rather than optimizing the response toward the uncertainty. In this regard, Branicki et al. (2018) indicate that the competitiveness of enterprises depends on their ability to identify and understand the detailed elements of risks so that appropriate strategies can be formulated. While the impacts of COVID‐19 are still unfolding, the successful adaptation to a complex situation and unforeseen future events requires the enhancement of the links between the planning and preparedness phases to reduce future risks as effectively as possible. Therefore, our analysis of risk perception and management strategies may provide useful insights that can be applied by managers and policymakers to make informed decisions and develop effective adaptation strategies to manage enterprise level and economy‐wide effects of the pandemic.

Furthermore, the present study contributes to the field of organizational resilience by taking a resource‐based view focused on the financial, physical, human, and organizational assets that identify and determine the risks that COVID‐19 poses to agri‐food SMEs and determines the ability of these enterprises to prepare for and respond to the pandemic. The organizational capability of an agri‐food SME can therefore be defined as a “firm's capacity to deploy resources for a desired end result” (Helfat & Lieberman, 2002). Therefore, the characteristics and resource base of an agri‐food SME would determine the extent to which it can generate a more proactive, informed, and effective stance to COVID‐19 risks.

## 3 DATA AND METHODS

### 3.1 Data and survey

A paper‐based questionnaire was designed to survey the risk perception and determinants in Egyptian agri‐food SMEs. The final version of the questionnaire was translated into Arabic and reviewed by three local researchers in the field of agribusiness administration and agricultural economics. Then, the questionnaire was pretested to five enterprises to ascertain the appropriateness of the order and flow of the questions and the clarity of the statements and to ensure that respondents interpreted and responded to these as intended.

At the beginning of each interview, the respondents were given a brief introduction to the study and were asked to answer questions regarding the potential consequences of the pandemic on the business activities of their agri‐food SMEs. The final questionnaire contained structured and open‐ended questions and consisted of seven sections to collect specific information on: (1) characteristics of the surveyed agri‐food SMEs; (2) perceived immediate impacts of COVID‐19 on agri‐food SMEs' business operations; (3) perceived long‐term impacts of the pandemic on the agri‐food SMEs; (4) sources of risk to SMEs' operations and performance; (5) SMEs' perception of various risk categories and total risks; (6) risk management strategies implemented by the surveyed agri‐food enterprises; and (7) supply chain opportunities emerging from the pandemic. With regard to COVID‐19 impacts, we included variables corresponding to the nine questions of the survey that were designed to capture the perceived impacts of the pandemic so far on agri‐food SMEs' sales and revenue, supply chain, cost of production, financial problems and cash flows, labor availability, and layoffs. Responses to these questions were coded on a Likert scale with six responses (see the questionnaire in the Supporting Information Material) from smaller to higher levels of impact, including the option of “unable to judge.” Finally, a categorical variable “*province*” was set to values between 1 and 6 if the agri‐food SME was located in Giza, Behaira, Kafr Al‐Shaikh, Cairo, Beni Suief, or Fayoum, respectively.

The sample consisted of randomly selected SMEs specializing in fresh fruit and vegetable sales both in the domestic and export markets. Within the survey, 234 agri‐food SMEs were contacted. Due to the partial lockdown and restrictions on people's mobility in Egypt during the pandemic, it was only possible to conduct interviews successfully with 166 agri‐food SMEs, equating to a response rate of 70.9%. Compared to previous similar studies carried out with Egyptian agri‐food enterprises (e.g., Abu Hatab et al., 2019; Hassan, 2016), both the sample size and response rate for this study was the highest.

Interviews were conducted from May 25 to June 21, 2020. Founders, managers, or key staff who make management decisions within the surveyed agri‐food SMEs were interviewed, as they were perceived to have the knowledge, experience, and perspective necessary to provide information regarding the impacts and risks experienced by their respective enterprises during the pandemic. The majority of the respondents (96.4%) were males, which reflects the male dominance in management positions in Egyptian agri‐food SMEs. Almost three‐quarters of the respondents were 35–45 years of age, and close to half of them have been in their current management positions for more than 5 years. About 58.4% of the respondents obtained university or postgraduate degrees, 27.7% completed postsecondary education, 11% completed primary or secondary education, and the remaining 3% were illiterate.

### 3.2 Measuring perceived risks

The term “risk,” refers to uncertainty, which originates from different events that can have either positive or negative consequences on business organizations (de Araújo Lima et al., 2020). However, in the context of corporate and business literature, risk traditionally describes the likelihood that an event, either expected or unexpected, may result in “unfavorable” effects on the business operations and require proper management (Knight, 2012). In the agribusiness literature, risk generally refers to “variations in net income from yield, price, and cost variability (Gabriel & Baker, 1980), and it has a long history of conceptual and empirical work focusing on risk identification and perception, risk measurement and the effectiveness of various risk management practices (e.g. Barry, 1984; Boehlje & Trede, 1977; Hardaker et al., 2015).” Within this literature, classification of risks according to their nature and influences is seen as an essential step to design and implement risk management strategies (Aimin, 2010; Schurle & Tholstrup, 1989).

As noted by Falkner and Hiebl (2015), risks for SMEs can be classified differently, implying that the motivation for choosing a classification must serve the purpose of research. In this study, previous research on the impacts of natural disasters and economic shocks to agricultural sectors and agri‐food SMEs and the specific factors that determine their postcrisis recovery, provided the basic framework for the design of our survey (e.g. Barry et al., 2001; Herbane, 2010; Pfeffer & Salancik, 1978; Sullivan‐Taylor & Branicki, 2011). An examination of this literature reveals that five broad risk‐source categories represent the main impact pathways through which risks posed by extreme events, such as the COVID‐19 pandemic, may be transmitted to SMEs. The first risk‐source category is “*supply chains*,” as extreme events disrupt SMEs' supply chains and often substantially reduce their production (e.g. Lu et al., 2020; Tokui et al., 2017). The second risk‐source category is “*sales and revenue*,” as SMEs, despite being more dynamic and opportunistic than larger enterprises, are vulnerable to significant fluctuations in demand for their goods and services (e.g., Cowling et al., 2015; M. A. U. Khan & Sayem, 2013). For instance, the government of Egypt implemented restrictions on human mobility during the months of May and June, 2020, which included a nationwide night‐time curfew, halting all public transportation between Egyptian governorates and closing all schools and universities, restaurants, churches, and mosques, and banned other large public gatherings (OECD, 2020b). Such human mobility restrictions significantly reduced private consumption and affected Egyptian SMEs' total monthly sales and revenues (CHF MCSE, 2020).

The third risk‐source category was denoted “labor,” as several studies have shown that dramatic demand and supply shocks resulting from pandemics and other extreme events can directly affect both labor supply and demand in SMEs (e.g., Lee & Warner, 2006; Varum & Rocha, 2013). The fourth risk‐source category was labeled “cost of production,” as unexpected shocks increase SMEs' business operating costs, raise direct and indirect costs relating to financial distress, and reduce their competitiveness (e.g. M. J. Khan et al., 2016; Yang et al., 2018). The final risk‐source category was labeled “*institutional and financial policies*,” as well‐designed and effectively implemented policies can contribute to capacity enhancement, thereby reducing risks associated with extreme events and promoting the ability of SMEs to adapt and recover (e.g., Pathak & Ahmad, 2018; Sullivan‐Taylor & Branicki, 2011).

A multidimensional model was then developed to measure the perception of risk within and across the five risk‐source categories following the approach by Lagerkvist et al. (2013). This approach introduces an intertemporal decision‐making component (i.e., delay or immediacy of exposure and impact) into the traditional model of perceived risk (i.e., weighting the probabilities of exposure and consequences). The interaction between timing and risks is supported by both theory and experimental results showing that delay and risk have their effects through a common underlying dimension (Keren & Roelofsma, 1995; Weber & Chapman, 2005). In a business setting, decision‐making in relation to, for example, the effects of a potential loss of skilled labor may actually be effects of risks entailed by the immediacy of occurrence. In addition, conversely, a delay in relation to a potential loss of skilled labor may not eliminate risks of exposure and consequences but rather increase the tolerance for such risks.

The questions included in our survey to measure the perceived risks among the surveyed agri‐food SMEs were designed from a tabular format in three steps to generate a weighted subjective assessment of perceived risks across these five categories. In the first step, the respondents were asked to rate each source of risk based on three different dimensions (severity [s], likelihood [l], and immediacy [t]). Specifically, the following statements were used to assess the three sub‐components of each source of risk.

▪ *Severity*: “How severe is the impact of this source on your enterprise's performance?” Respondents were instructed to freely assign a score on a 0–10 scale as appropriate with the following alternatives: “not at all a problem” (0), “minor problem” (2.5), “moderate problem” (5), “serious problem” (7.5), and “very serious problem” (10).
▪ *Likelihood*: “What do you think the likelihood is that your enterprise will be affected by the source of the risk?” with the following alternatives: “none” (0), “very low” (1), “low” (2.5), “medium” (5), “high” (7.5), and “very high” (10).
▪ *Immediacy of impact*: “When in the future do you think you will notice any harmful impact of the source of risk to your enterprise's performance?” with the following alternatives: “immediately” (10), “within a few weeks” (8), “within 3 months” (4), “after a longer time” (end of 2020) (1), “and not at all” (0).

For each risk source (*i*), and allowing for the fact that severity, likelihood and immediacy are interrelated, the perceived risk was calculated as $r_{i}=\left(s_{i}\times l_{i}\times t_{i}\right)/10$ so as to have a per‐item risk score on the interval (0, 100). To illustrate this, consider the assessment of the three components (*s*, *l*, and *t*) of perceived risk for the risk source “Decrease in total value of monthly domestic sales” within the risk dimension “Revenue/sales.” The perceived risk would then be scored as 7.5 [=(2.5 × 7.5 × 4)/10] if the triple response of a given respondent would be that: severity = 2.5 (minor problem), likelihood = 7.5 (high), and; *t* = 4 (after a longer time, i.e., later than in 3 months).

Proceeding to Step 2, the respondents were asked to rate the relative importance of each risk source (*w_i_*) within each risk dimension respectively (e.g., the importance of “decrease of monthly domestic sales” relative to the other three risk sources with the revenue/sales dimension), resulting in relative weights within each dimension adding up to 100%. Based on this input, the weighted risk score per dimension (*k* = 1,. .5) was calculated as $rd_{k}=\sum_{i=1}^{n}w_{i}r_{i}$ where *n* denotes the number of risk sources with the dimension.

Finally, in Step 3, respondents were presented with the five dimensions of risk and asked, “Overall, what is the relative importance of these risks to your agri‐food SME from these five different sources?” Specifically, respondents were given 100 points to distribute across the five risk dimensions based on a constant‐sum scaling of feature importance (*I*_k_) and were permitted to adjust and verify the distribution of their marks before completing this step. Based on this input, the final total risk score was calculated as $\mathrm{tot}\mathrm{risk}=\sum_{k=1}^{k=5}I_{k}rd_{k}$ with a potential minimum of zero and a maximum of 100.

To sum up, this three‐step approach for measuring perceived COVID‐19 risks among the surveyed agri‐food SMEs allowed for a distribution‐free characterization of heterogeneity in the risk perceptions (Steps 1 and 2). It also ensured that the respondents were familiar with all risk sources, had thoroughly considered the extent of each source of risk, and demarked and evaluated the overall relative importance of the five risk categories. Table 2 shows the sources of risk that were used to assess the perceived risks associated with the COVID‐19 pandemic among the interviewed agri‐food SMEs.

### 3.3 Conditional inference

Recursive partitioning methods for classification and regression is established as valid alternative to parametric methods (Steingrimsson et al., 2018). For the purpose of this study, the nonparametric conditional regression tree approach from Hothorn et al. (2006), a machine‐learning algorithm, was adopted and used in the analysis. Specifically, this approach was employed to analyze how perceived business risks in relation to COVID‐19 are associated with a large number of potentially relevant covariates (some of which might be endogenous) to statistically identify those with both the highest relevance and the largest explanatory power. This approach splits dataset so that the dependent variable is separated into as many statistically distinct subgroups as possible. The algorithm then generates, from the set of potential covariates, a nested structure of subdatasets until no further statistically significant splits (according to Bonferroni adjusted *p* values) can be identified. Differently, from directional predictive analysis, the HHZ approach then serves to isolate specific levels of covariates and reveal how they, if at all, convert to different subgroups of total risk. Furthermore, some of the covariates might not be involved in any split, which means the tree‐based method automatically performs the variable selection. The HHZ approach has been extensively used for conditional inference within several disciplines. Recent applications within business research include consumer branding (Schivinski, 2019) as well as innovation and technology transfer (Guerzoni et al., 2020).

In the HHZ approach, covariate selection and the splitting procedure was set to test the omnibus null hypothesis of independence between perceived risk and any of the potential covariates at *α* = .05 (based on multiplicity adjusted *p* values) and to stop if this hypothesis could not be rejected. Otherwise, the covariate with the strongest association with the perceived risk was identified together with a binary split based on a partial null hypothesis for the single covariate and the risk measure. This procedure was then repeated until the omnibus null hypothesis could not be rejected further. Estimation of the HHZ model was performed using the CTree module in the R package *partykit* (Hothorn & Zeileis, 2015) within the R network package (R Development Core Team, 2013).

For the conditional inference analysis, we separated perceived risk by agri‐food SMEs with fruit and vegetables export from other types of primary production for export. That is, we introduced the variable “*specialization export fruits*“ (*n* = 58), which took the value 1 if the primary production for export was related to fresh fruit) and the variable “*specialization export vegetables*” (*n* = 38), which took the value 1 if the primary production was related to fresh vegetables). Moreover, we coded the variable “*Foreign sales*” (share) as the ratio of export sales to total sales. Furthermore, a categorical variable was set to differentiate the primary destination of exports to either (1) Europe (*n* = 55), or (2) Arab Gulf (*n* = 76), from other regions (0).

### 3.4 Characteristics of the sample agri‐food SMEs

Table 1 summarizes background information and the main characteristics of the surveyed agri‐food SMEs. In terms of ownership structure, the majority of the enterprises (85%) have 100% domestic ownership, only 2% have 100% foreign investment, and the remaining 13% have joint foreign and domestic investment. As per 2019, the agri‐food SMEs in our sample had total assets varying from less than EGP 3 million (38.5%) to more than EGP 10 million (14%). The surveyed enterprises have varied levels of experiences in the agri‐food business: 11% have been operating for over 20 years, 8% for 15–20 years, 32% for 10–15 years, and 49% were relatively new to the business with less than 10 years in agri‐food activities. The majority of the sample (87%) has only one branch, whereas the remaining enterprises have two or more branches. About half of the surveyed agri‐food SMEs were characterized by a relatively small number of employees (less than 20), 40% have between 20 and 50 employees, and 10% have more than 50 employees. The relatively small number of employees within the surveyed enterprises can be explained by the fact that Egyptian agri‐food SMEs customarily rely on seasonal workers and other enterprises to outsource or subcontract specific services during peak seasons (Abu Hatab et al., 2019).

**Table 1:** Sample characteristics (*n* = 166)

Characteristics	Categories	Frequency	Percent of the sample
Years in business	<5	40	24.10
	5 to <10	41	24.70
	10 to <15	53	31.93
	15 to <20	14	8.43
	≥20	18	10.83
Ownership type	Local investment	141	84.94
	Foreign investment	3	1.81
	Joint investment	22	13.25
Number of employees	<10	24	14.46
	10 ‐<20	68	40.96
	20 ‐<50	67	40.46
	>50	7	4.22
Number of branches	1	145	86.75
	2	5	3.01
	≥3	16	10.24
Exports/total sales (%)	<30	42	25.31
	30 ‐<50	14	8.43
	50 ‐<70	10	6.02
	≥70	100	60.24
Total assets in 2019 (EGP million)	<3	64	38.55
	3 ‐<5	59	35.54
	5 ‐<10	20	12.05
	≥10	23	13.85
Turnover in 2019 (million EGP)	<3	73	43.98
	3 ‐<5	40	24.10
	5 ‐<10	35	21.08
	≥10	18	10.84
Region	Rural	40	24.10
	Peri‐Urban	60	36.14
	Urban	47	28.31
	Industrial Zone	19	11.45
Province	Giza	14	8.43
	Nobaria	35	21.08
	Kafr AL‐Shaikh	20	12.05
	Cairo	27	16.27
	Beni Suief	34	20.48
	Fayoum	36	21.69

*Note*: Average of US dollar to EGP exchange rate in 2019 = 16.814 EGP (Central Bank of Egypt).

*Source*: Survey results.

**Figure 1:**
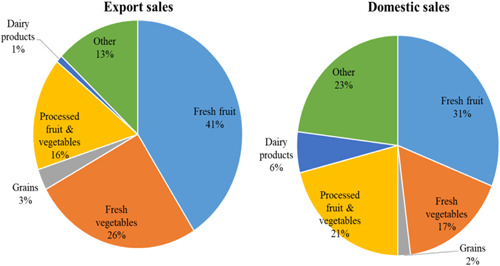
Percent distribution of main categories of domestic and foreign sales of the surveyed agri‐food small and medium agri‐food enterprises. *Source*: survey results [Color figure can be viewed at wileyonlinelibrary.com]

The summary statistics in Table 1 indicate that enterprises in our sample are export‐oriented, with 60% having an export to total sales ratio of 70% or more. The remaining enterprises have export/total sales ratios ranging between 30% and 70% (14% of the sample) and less than 30% (25% of the sample). The agri‐food SMEs in our sample specialize mainly in fruit and vegetables and operate both on the domestic and international markets (Figure 1). On the domestic market, the main categories for the most frequently sold products are fresh fruit (31% including grapes, oranges, and other citrus), processed fruits and vegetables (21%, including fruit juice, frozen vegetables, and pickles), and fresh vegetables (17%, including onion, tomato, and potato). In addition to these products, some of the interviewed agri‐food SMEs indicated that they also sell dairy products (6%), grains (2%), and other products (22%), such as seeds and fertilizers, medicinal and aromatic plants, and animal feed.

On the export market, the agri‐food SMEs surveyed were more specialized, with fresh fruit (41%, including oranges and citrus) and fresh vegetables (26% including potato, onion, and grapes) representing two‐thirds of the total foreign‐market sales. Around 16% of the agri‐food SMEs export processed fruit and vegetables, such as juice, frozen vegetables, and dried onion, and an almost equal percentage of them export grains, dairy, and other products (Figure 1). Furthermore, the survey results revealed that the Arabian markets (mainly Arab Gulf States), followed by the EU markets, represent the main export destinations, accounting for 46% and 33% of the total exported agri‐food commodities from the surveyed agri‐food SMEs, respectively.

With regard to turnover, the results reveal that 44% of the enterprises had annual sales in 2019 of less than EGP 3 million, whereas 24% had annual sales between EGP 3 to 5 million and 32% had annual sales of more than 5 million. Table 2 presents descriptive statistics for selected characteristics and performance indicators for the surveyed enterprises. One concern that should be highlighted is that estimates regarding turnover and total assets, in particular, tend to be underestimated, and as such are only reliable to the degree that the managers at the agri‐food SMEs provided accurate information.

**Table 2:** Descriptive statistics of selected characteristics and performance indicators of the surveyed enterprises

	Years in business	Number of employees	Percent of export/total sales	Total assets Million EGP	Turnover Million EGP
Mean	9.78	20.79	63.79	2.14	2.81
SD	7.25	12.78	38.87	1.32	1.26
Minimum	1	3	0	1	1
Maximum	54	90	100	6	5
Percentiles					
25	5	12	25	1	2
50	10	18	80	2	3
76	12	26.25	100	3	4
*N*	166	166	166	166	166

**Figure 2:**
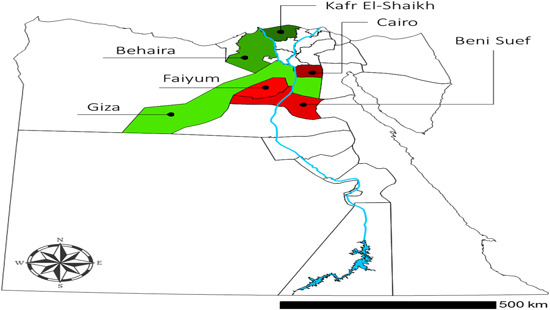
Study areas. *Note*: Areas highlighted in green represent newly reclaimed lands and desert areas (NRLDA), whereas those highlighted in red represent old lands in the Nile delta areas (OLNDA) [Color figure can be viewed at wileyonlinelibrary.com]

The spatial distribution of the sample reveals that around 42% of the surveyed enterprises are located in the “new lands” reclaimed in the Western desert along the Alexandria‐Cairo desert highway in Giza and Behaira and in the Nile Delta in Kafr Al‐Shaikh. The rest of the sample is located in the “old lands” along the Nile Delta in Cairo (16%), Beni Suief (20%), and Fayoum (22%). Within these six provinces, around one‐quarter (24%) of the surveyed enterprises are located in rural areas, 36% in periurban areas, 28% in urban areas, and 11% in industrial zones. Figure 2 below displays the geographic locations of each site.

## 4 RESULTS

### 4.1 COVID‐19 induced challenges to the surveyed agri‐food SMEs

A module consisting of several questions was included in the survey to capture the major challenges encountered by the surveyed agri‐food SMEs due to the COVID‐19 pandemic and their impacts on their businesses. Table 3 below shows that the pandemic caused a diverse set of challenges for agri‐food SMEs, with a reduction in sales being the most frequently mentioned challenge (41% of the sample). Around 27% of the surveyed enterprises indicated that the pandemic caused significant upstream and downstream chain disruptions and made it difficult to meet their contractual obligations. Other challenges indicated by the respondents were mentioned less frequently, and they tend to be consequences of the pandemic's effects on the agri‐food SMEs' supply chains and sales, including increased difficulty of obtaining financing (12%) and subsequent inability to pay existing loan installments (7%), payroll (2.4%), and rent, and other invoices (2%).

**Table 3:** Main challenges caused by the COVID‐19 pandemic to the business of agri‐food SMEs

Challenges	Frequency	Percent of the enterprises
Disruption of supply chain and logistics	24	14.46
Inability to deliver existing orders	21	12.65
Increased difficulty in obtaining financing	20	12.05
Inability to pay rent and other invoices	4	2.40
Problems with paying off loan installments	11	6.63
Reduction in market demand, sales, and revenue	68	40.96
Inability to pay employees' salaries and wages	4	2.41
Other problems	14	8.43
Total	166	100

*Note*: The question in the survey was “At this point, what are the main business problems your enterprise is currently facing due to the pandemic?”

*Source*: Survey results.

Table 4 shows percentage distributions of responses to the questions of how the cost of production and the total revenue of the surveyed enterprises in April 2020 compared to these of April 2019. Close to 60% of the enterprises indicated that compared to April 2019, their total revenue in April 2020 decreased by less than or equal to 10% (27%) or more than 10% (32%). In contrast, 10% of the surveyed agri‐food SMEs believed that their total revenue increased by less than or equal to 10%, whereas another 16% of the enterprises believed that their total revenue increased by more than 10%. The total revenue of the remaining 15% of the sample remained unchanged or the respondents were unable to judge.

**Table 4:** Impacts of the COVID‐19 pandemic on agri‐food SMEs' cost of production and revenue

Impact	Cost of production^a^		Revenue^b^	
	Frequency	Sample (%)	Frequency	Sample (%)
Decrease of less than or equal to 10%	7	4.22	45	27.11
Decrease of more than 10%	16	13.86	53	31.93
Increase by more than 10%	80	48.19	26	15.66
Increase, but less than or equal to 10%	45	27.11	17	10.24
Same as last year	15	9.04	23	13.86
Unable to judge	3	1.81	2	1.20
Total	166	100	166	100

^a^ The question in the survey was “At this point, how would you evaluate the cost of your company's raw materials and total operating costs in April 2020 compared to April 2019?”

^b^ The question in the survey was “At this point, how does the total revenue of your company during April 2020 compare to April 2019?”

With regard to the effects of the pandemic on the enterprises' production costs, Table 4 shows that three‐quarters of the surveyed agri‐food SMEs experienced increases in production costs, with 64% of these enterprises registering increases of more than 10%. Only 13% of the surveyed enterprises reported decreases in their production costs between April 2019 and April 2020, whereas 9% experienced no changes in their production costs. In connection with this, it is apparent that layoffs was one of the main strategies on which agri‐food SMEs in our sample relied to minimize their operating costs. As shown in Table 5, more than half of the enterprises surveyed considered layoffs or have already laid off up to 30% of their workforce.

**Table 5:** The impact of the COVID‐19 pandemic on layoff of the surveyed agri‐food SMEs' labor

Layoffs	Frequency	Percent of the sample
1–10	73	43.98
11–20	17	10.24
21–30	4	2.41
More than 30	1	0.60
Unable to judge	26	15.66
No layoffs	45	27.11
Total	166	100

*Note*: The question in the survey was “If your company currently considering layoffs, or has already done some because of the epidemic, what number of staff are you expecting to (or have already) cut?”

**Figure 3:**
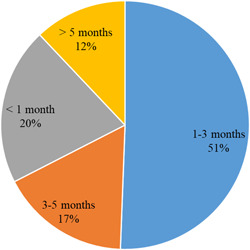
The impact of the COVID‐19 pandemic on the surveyed agri‐food small and medium agri‐food enterprises. *Note*: the question in the survey was “How long can your company's current cash flow maintain the company's operation?” *Source*: survey results [Color figure can be viewed at wileyonlinelibrary.com]

On a question concerning the duration that the SMEs could maintain operations given current cash flows, 20% estimated this period as less than 1 month (Figure 3). About half of the sample reported that their cash flow was sufficient to maintain their current production and pay financial commitments including dividends for a period between 1 and 3 months. Other enterprises estimated this duration between 3 and 5 months (17%) or more than 5 months (12%). Finally, only 19% of the enterprises reported that they had written, pre‐established guidelines for risk management, whereas the majority of the surveyed agri‐food SMEs operate mostly in a reactive mode. The absence of proactive and preventative risk‐management strategies may lead to lack of preparedness among the surveyed SMEs and explains their vulnerability to the impacts of COVID‐19.

### 4.2 Perceived importance of different sources of COVID‐19 risks and overall risk score

Table 6 provides descriptive statistics of the severity, likelihood, and immediacy of different sources of risks associated with COVID‐19 as perceived by the surveyed enterprises. In particular, the results reveal that respondents regarded the effect of immediacy for “labor,” “sales and revenue,” and “supply chain” risks to be greater than the impact of severity and likelihood.

**Table 6:** Perceived importance and evaluative scores for different risk sources as perceived by interviewed agri‐food SMEs

Risk categories	Source of risk	Dimensions of risk sources					
		Severity		Likelihood		Immediacy	
		Median	Mean	Median	Mean	Median	Mean
Sales and revenue	Decreased value of monthly domestic sales	5	4.6	7.5	5.1	8	5.1
	Decreased value of monthly export sales	7.5	7.0	7.5	7.0	8	6.6
	Delays & reduced collection of receivables	7.5	7.5	7.5	7.8	8	8.0
	Reduced purchasing power of consumers	7.5	7.3	7.5	7.2	8	7.1
Supply chain	Restrictions caused shipments grounded at ports	7.5	6.8	7.5	7.4	7.5	7
	Restrictions on transportation and disruptions in distribution channels to markets	5	4.3	5	4.2	4	5.1
	Rejection of shipments by importer/retailer	7.5	5.8	7.5	6.3	8	5.5
	Failure to deliver contracted sales to partners	7.5	6.7	7.5	7.0	8	7.3
	Delayed port operations	5	6.1	7.5	6.3	8	7.3
	Shortage in quantities of agricultural commodities to meet demand	7.5	6.1	7.5	6.1	8	7.5
	Reduced quality of agricultural commodities	5	5.9	7.5	6.4	8	7.1
Labor	Plummeting employee productivity: employees are unable to commute to work	5	5.5	5	5.6	8	7.0
	Loss of skilled labor	5	4.7	5	4.8	8	6.6
	Reduction in the number of working days	5	5.9	7.5	6.0	8	7.1
	High rates of work absenteeism	7.5	6.0	7.5	6.5	8	7.2
Cost of production	The cost of commodities sold by the SME increased	10	8.7	10	8.9	10	8.8
	SMEs cannot afford investments for market and technological development	0	1.9	0	2.1	0	1.7
	The cost of inputs increased	7.5	8.0	7.5	8.2	8	8.1
	Increased losses and waste	5	6.7	7.5	6.9	8	7.9
	Cost of implementing preventative measures at the workplace	7.5	6.4	7.5	6.5	8	8.0
Institutional and financial policies	Reduced capacity of public and private institutions to provide services to SMEs	5	5.9	5	6.2	4	6.2
	Policy uncertainty, w.r.t., corporate tax	5	6.0	7.5	6.3	4	5.8
	Policy uncertainty with regard to cutting employee numbers and salaries	5	5.6	7.5	6.0	4	5.7
	Policy uncertainty with regard to (central) bank's interest rate policies	5	5.5	5	5.7	4	4.7
	Economic recession or political instability	5	5.7	5	5.8	4	4.5
	Difficulty accessing emergency support introduced by government departments.	7.5	6.9	7.5	7.1	8	6.1
	Increased cost of obtaining loans	5	4.9	7.5	5.1	4	4.6
	Banks and financial sector organizations are unwilling to provide credit to SMEs	5	4.8	5	4.8	4	4.7
	Inability to pay back my loans	5	4.8	5	5.1	4	4.5

*Source*: survey results.

The ratings for the effects of severity and likelihood were nearly identical for these three risk categories. The higher ratings for the effect of immediacy of risks for these two categories, compared to the effects of severity and likelihood, could be explained by the major short‐term disruptions caused by COVID‐19 in agri‐food supply chains, and the functioning of domestic agricultural markets as well as the subsequent, sudden drop in domestic sales, and export sales for the surveyed enterprises. This finding is in concert with the findings of Abass et al. (2019) and Lagerkvist et al. (2013), which indicate that for short‐term sources of risk, the effect of immediacy is greater than the impact of severity. However, for sources of risk with longer term consequences, the impacts may not be immediate but could delay and manifest after a long time, and thus the effects of severity and impact can be much higher.

With regard to risks related to input and output costs, the results reveal that the effects of severity, likelihood, and immediacy in relation to the increased cost of agri‐food commodities sold by the agri‐food SMEs were equal and were rated highest among other sources of risk across all risk categories. Interestingly, the respondents generally considered the effect of likelihood for risks associated with government policies to be greater than the impacts of immediacy and severity. Most of the government policies concerning COVID‐19 were unpredictable (e.g., corporate tax and interest rate), temporary, and many of them were loosely implemented in rural and periurban areas where the majority of agri‐food SMEs are located (e.g., preventative measures at the workplace). Moreover, many of these institutional policies have gradually been relaxed or abandoned (e.g., emergency support schemes). Thus, low ratings for the effects of immediacy and severity are expected. In connection with this, the results revealed the effects of financial‐related sources of risk were generally perceived as less important for the surveyed agri‐food SMEs. Previous studies have shown that financial services provided to Egyptian agri‐food SMEs are poorly operated and underfunded and that the business of these enterprises is constrained by the unwillingness of financial institutions to provide credit and other services to this segment (e.g., Abu Hatab & Hess, 2013). Thus, the fact that agri‐food SMEs are traditionally disadvantaged, disengaged, and/or excluded by Egyptian financial institutions may explain the low ratings related to financial sources of risk.

Table 7 presents the results of the Pearson correlation coefficient between risk dimensions. All correlation coefficients were positive. In general, the correlation between likelihood and severity was the strongest, with a mean of .75 and coefficients ranging between .42 and .93. Correlation coefficients between severity and immediacy and between severity and likelihood were quite similar and ranged between .12 and .77 for both risk dimensions.

**Table 7:** Person's correlation between risk dimensions

Source	Person's correlation		
	Severity‐likelihood	Severity‐immediacy	Likelihood‐immediacy
Decreased value of monthly domestic sales	0.88	0.68	0.72
Decreased value of monthly export sales	0.90	0.74	0.76
Delays and reduced collection of receivables	0.52	*0.16*	0.25
Reduced purchasing power of consumers	0.61	0.39	0.33
Shipment of fresh produce grounded at airports and ports because travel has stopped	0.84	0.70	0.71
Restrictions on transportation and disruptions in distribution channels to markets	0.80	0.54	0.58
Rejection of shipments by importer/retailer	0.42	0.77	0.47
Failure to deliver contracted sales to partners	0.55	0.41	0.37
Delayed port operations	0.72	0.32	0.26
Shortage in quantities of agricultural commodities to meet the demands	0.77	0.23	0.26
Reduced quality of agricultural commodities	0.81	*0.16*	*0.19*
Plummeting Employee Productivity: employees are unable to commute to work	0.73	0.41	0.39
loss of skilled labor as experienced employees with valuable information and knowledge and/or contacts leave the firm	0.75	0.38	0.30
Reduction in the number of working days	0.77	0.53	0.50
High rates of work absenteeism	0.76	0.59	0.65
The cost of commodities sold by the firm increased	0.70	0.34	0.34
SMEs cannot afford investments for market and technological development	0.93	0.70	0.72
The cost of inputs and (e.g. labor, fertilizer) increased	0.67	0.40	0.39
Increased the losses and waste	0.74	*0.20*	*0.14*
Cost of implementing preventative measures at the workplace	0.76	0.39	0.47
Reduced capacity of public and private institutions to provide services to SMEs	0.79	0.42	0.43
Policy uncertainty with regard to corporate tax	0.74	0.25	0.21
Policy uncertainty with regard to cutting employees' numbers and salaries	0.67	0.29	0.23
Policy uncertainty with regard to (central) bank's interest rate policies	0.84	0.39	0.38
Economic recession or political instability	0.78	*0.12*	*0.17*
Difficulty to access emergency support introduced by government departments	0.86	0.58	0.53
Increased cost of obtaining loans	0.83	0.44	0.46
Banks and financial sector organizations are unwilling to provide credit to SMEs	0.81	0.37	0.37
Inability to pay back my loans	0.88	0.49	0.51
*Median*	0.77	0.40	0.39
*Mean*	0.75	0.43	0.42
*Max*	0.93	0.77	0.76
*Min*	0.42	0.12	0.14

*Note*: Estimates are significant at the 0.01 significance level, except for the ones in italic.

*Source*: survey results.

The severity components were strongly correlated with the likelihood for almost all risk categories, particularly for the “*institutional and financial policy risks*,” “*cost of production*,” and “*labor*.” The correlation between severity and immediacy dimensions of risk was relatively stronger for “*sales and revenue*” and “labor” as well as sales and revenue risks. Furthermore, Table 7 shows that the immediacy and likelihood of risk were above average or below average correlated for all risk categories, with items within the “*sales and revenue*” category having the highest correlation coefficients.

Finally, Table 8 summarizes the overall weights and the relative importance of the main categories of risk sources. In general, the results indicate that changes in sales and revenue represent the most important pathway through which the effects of the COVID‐9 pandemic were transmitted to the surveyed agri‐food SMEs. Risks originating from supply chains disruptions were ranked the second most important channel for the effects of the pandemic on the enterprises. Notably, the overall importance of other risk categories, particularly those related to labor and institutional polices, was relatively low.

**Table 8:** Summary statistics for the main risk categories and total weighted risk

Risk categories	Weighted risk				Relative risk importance			
	*Minimum*	*Maximum*	*Mean*	*SD*	*Minimum*	*Maximum*	*Mean*	*SD*
Sales and revenue	5.1	100.0	49.7	22.3	0	60	29.1	8.7
Supply chain	0.0	100.0	40.2	21.5	0	50	23.3	8.6
Labor	0.5	91.3	31.9	22.9	0	40	13.6	7.7
Cost of production	5.3	100.0	53.8	21.2	5	40	18.6	7.7
Institutional and financial policies	0.6	87.4	30.5	25.9	0	45	15.4	7.7
Total weighted risk score	12.4	92.5	43.6	17.7				

### 4.3 Determinants of perceived risk among the surveyed agri‐food SMEs

**Figure 4:**
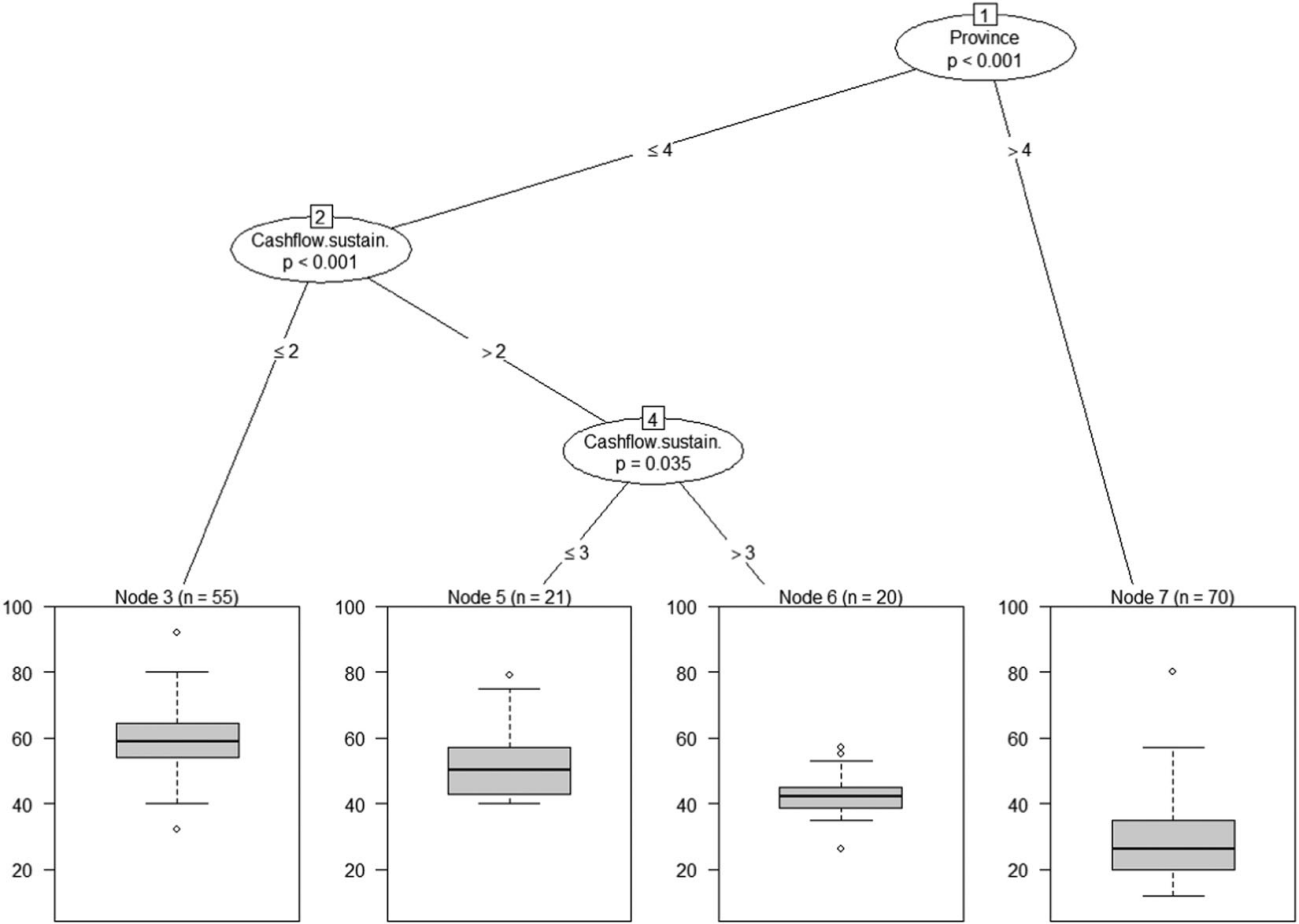
Probabilistic conditional binary recursive inference for total risk and p‐values and associated split criteria between total risk and the full set of explanatory variables related to current COVID‐19 challenges. *Note*: Box plots show the splits so that the dependent variable (end‐nodes) is separated into as many statistically distinct subgroups as possible. End‐nodes shows the distribution of total perceived risk per subgroup(median, 25% and 75% quartile) and the extended bars indicate the highest and lowest points in the data. The algorithm then generates, from the set of covariates, a nested structure of subdatasets until no further statistically significant splits (according to Bonferroni adjusted *p* values) can be identified. Covariates included in the analysis were: Province, Region, Cashflow.sustain (cash‐flow coverage periods), Revenue2020 (change in total revenue of agri‐food SMEs in April 2020 compared to that of April 2019), Cost2020 (change in production cost in April 2020 compared to that of April 2019), Mainbusinesschallenge (main business challenges, see Table 3), and Layoffs (impact of the pandemic on labor layoff at the surveyed agri‐food SMEs). SME, small and medium agri‐food enterprise

**Figure 5:**
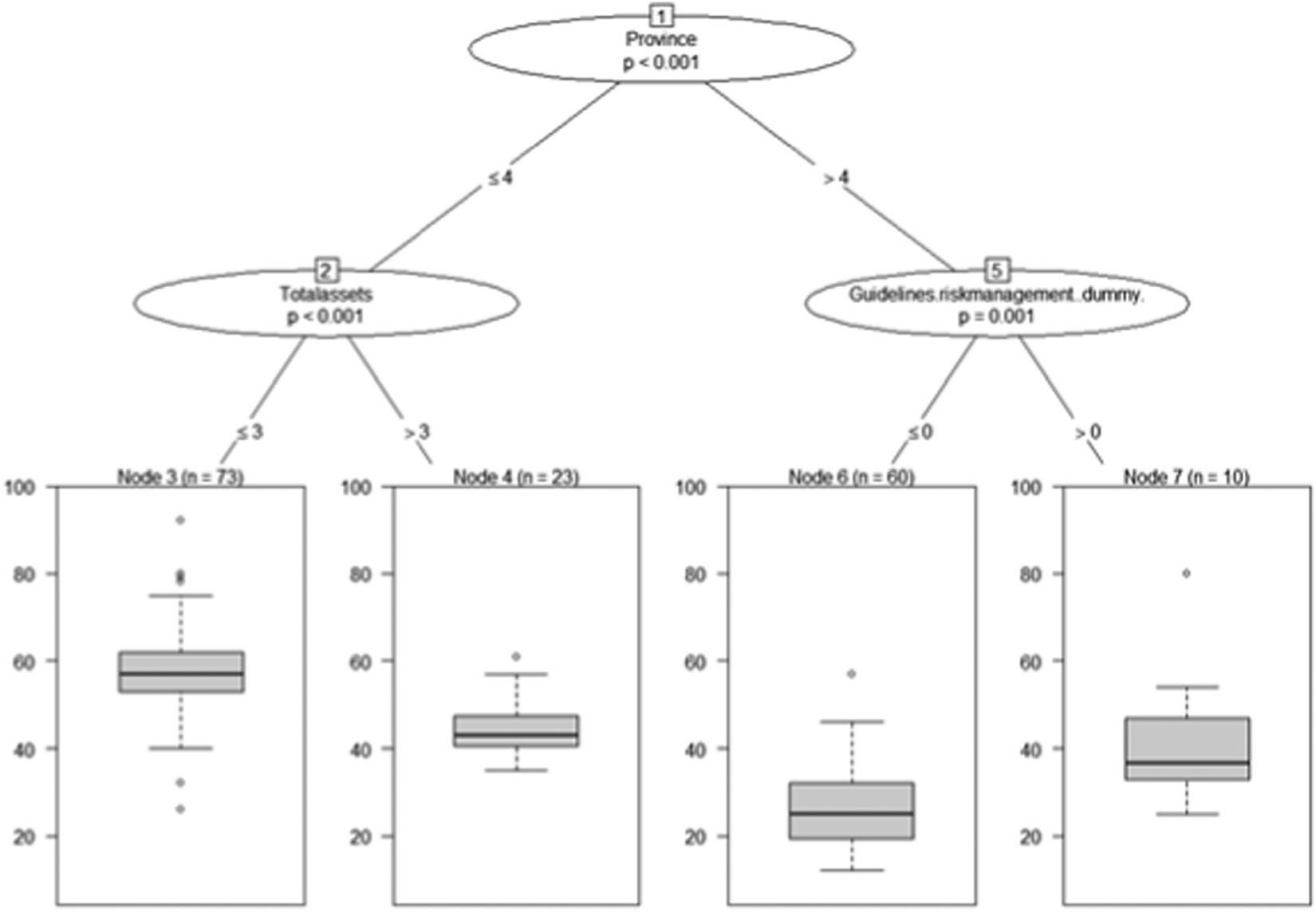
Probabilistic conditional binary recursive inference for total risk (*p* values and associated split criteria between total risk and the full set of explanatory variables related to exposure to geographic location and SMEs' characteristics. *Note*: See Figure 4 for computational details. Covariates included were: Province, Region, Years in business (the number of years the SME has been in business), Employees.number (the total number of employees), Foreignsales.share (the ratio of export sales to agri‐food SMEs' total sales), Specialization.domesticfruits (SMEs' specialization in the domestic sales of fruit), Specialization.exportfruits (SMEs' specialization in the export of fruit), Specialization.exportfreshvegetables (SMEs' specialization in the export of vegetables), Primaryexportmarket (SMEs' main export destination), Total assets (the value of SMEs' total sales), Turnover (the value of SMEs' annual sales), Qualitycertified.dummy (a dummy variable that takes one, if the agri‐food SME was certified for a quality standard system), Type of certificate (the quality standard system for which the SME is certified, e.g., GlobalGAP), and Guidelines.riskmanagement.dummy (a dummy variable that takes one, if the agri‐food SME has internal guidelines for risk management). SME, small and medium agri‐food enterprise

**Figure 6:**
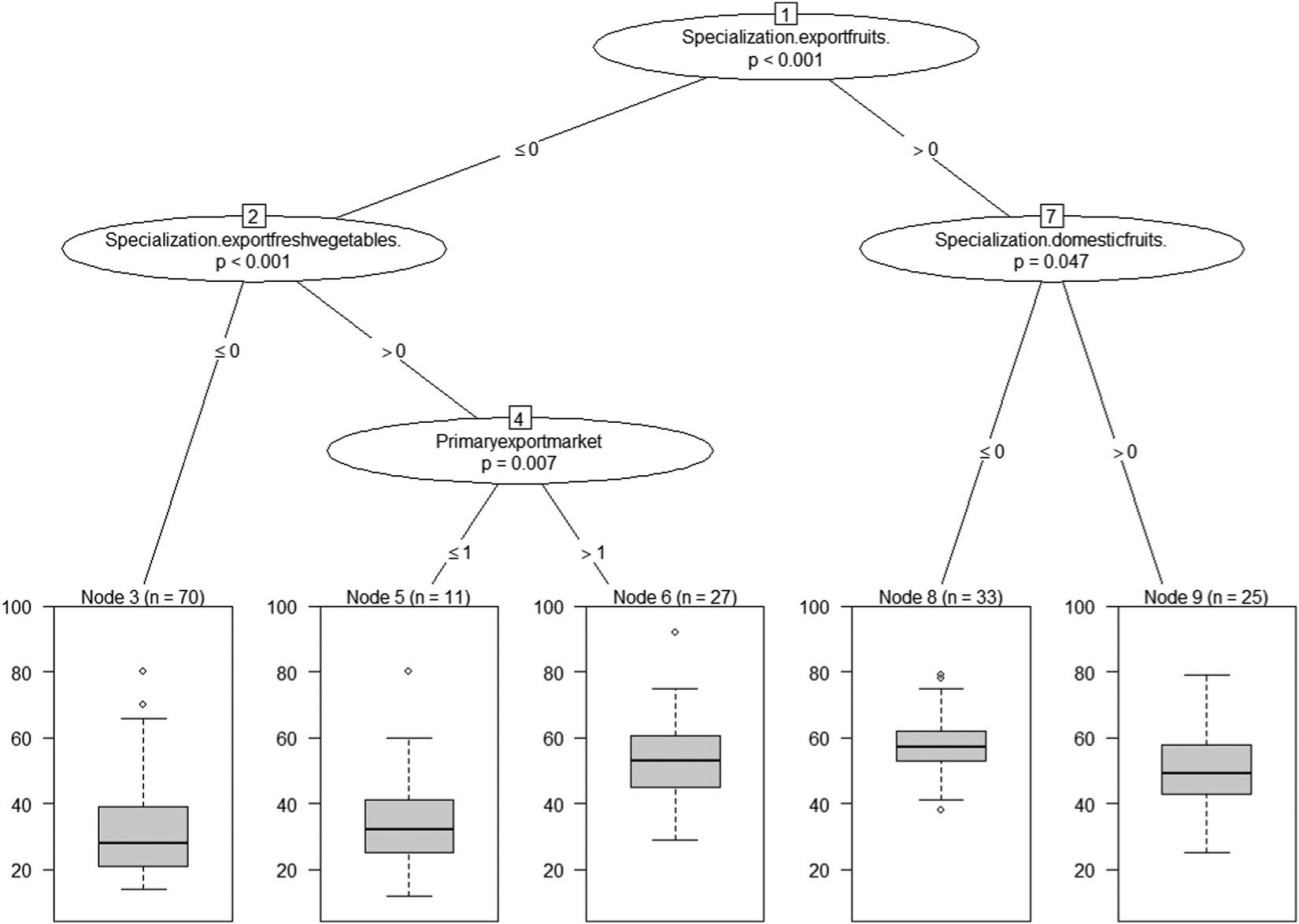
Probabilistic conditional binary recursive inference for total risk. *p* values and associated split criteria between total risk and the full set of explanatory variables related to exposure to market orientation. *Note*: See Figure 4 for computational details and Figure 5 for the definition of covariates

In this section, we statistically identified covariates with both the highest relevance and the largest explanatory power for the total risk perceived by the surveyed agri‐food SMEs using three main dimensions of the risk related to the Covid‐19 pandemic, namely: (i) current COVID‐19 challenges; (ii) SMEs' geographic location and characteristics; (iii) and market orientation. Figures 4, 5–6 display the box plots of the total risk score (end nodes), which represents the dependent variable in the estimated HHZ model. With regard to the first dimension of our analysis, “*Current COVID‐19 challenges*.” Figure 4 reveals that SMEs' location is the first and most significant split determinant, dividing the total risk score into two spatially distinct subsets: agri‐food SMEs in newly reclaimed lands and desert areas (NRLDA) and agri‐food SMEs in old lands in the Nile delta areas (OLNDA). Being located in NRLDA was related to “Node 7,” which corresponds to a subsample of *n* = 70 agri‐food SMEs. For agri‐food SMEs located in OLNDA, further splits into additional subsamples proved significant, where the duration up to which the current cash flow of the SMEs can maintain their operation “cash flow” was the next most significant determinant.

Specifically, this determinant split the sample into two subsets of agri‐food SMEs with shorter (less than 3 months) and longer (more than 3 months) cash‐flow coverage periods. On the one hand, the subsample of agri‐food SMEs with less than 3 months of cash‐flow coverage period was related to “Node 3” and consisted of 55 agri‐food SMEs. On the other, the subsample of agri‐food SMEs with more than 3 months of cash‐flow coverage period was further divided by the same variable (cash‐flow coverage period) into two sub‐sets: agri‐food SMEs with cash‐flow coverage period of up to 5 months, and agri‐food SMEs with cash‐flow coverage period of more than 5 months.

With regard to the second dimension of our analysis geographic location and SMEs' characteristics, Figure 5 shows that the province in which the agri‐food SMEs are located (*province*) was the most significant split determinant, dividing the *total risk score* into two subsets: agri‐food SMEs located in NRLDA and agri‐food SMEs located in OLNDA. For the NRLDA subsample, “*total assets*” represented the most significant determinant of total risk score. With regard to OLNDA agri‐food SMEs, the results revealed that having “*internal guidelines for risk management*” was the most significant determinant of total risk score. Interestingly, enterprises that have risk management strategies perceived higher risks during the COVID‐19 pandemic compared to those that do not have such strategies. Overall, the results in Figure 5 strongly reaffirm the results in Figure 4, confirming that total assets and cash holdings function as a buffer against potential adverse shocks and reduce the probability of experiencing financial distress.

In relation to the third dimension of the analysis “*exposure to market orientation*,” Figure 6 show that “*specialization in fruit export*” was the first and most important split determinant that broke the sample into two subsets: “fruit exporting SMEs” on the one hand and “nonfruit exporting SMEs” on the other. For fruit exporting enterprises, specialization in the domestic sales of fruit was the most significant determinant. That is, agri‐food SMEs that operate in both the domestic and international markets face less risks compared to those who sell exclusively in the international market. For nonfruit exporting SMEs, specialization in the “*export of fresh vegetables*” was the most significant determinant, dividing the total risk score into two subsets: “vegetable exporters” and “nonvegetable exporters.” For vegetable‐exporting enterprises, further splits into additional subsamples proved significant with the *primary export market* being the most significant determinant. Interestingly, the results suggest that agri‐food SMEs whose main export destination is Arab countries faced significantly higher COVID‐19‐induced risks compared to enterprises with main export market being the EU or other destinations.

## 5 DISCUSSION AND CONCLUDING REMARKS

Although coping with extreme events and uncertainties has been a frequent topic in organizational theory, little research has been undertaken to investigate how SMEs in general and agri‐food SMEs in particular deal with uncertainties created by extreme events and natural hazards in developing countries. Previous studies pointed out that risk perception and risk management approaches widely differ by a range of organizational factors and that understanding these aspects in relation to organizational size, particularly how SMEs perceive and cope with extreme events, is under‐examined (Sullivan‐Taylor & Branicki, 2011). This study was a step toward addressing this gap in terms of both theory and practice. Specifically, in our empirical analysis, risks associated with the COVID‐19 pandemic were viewed as an unprecedented situation that posed significant risks and led to high levels of uncertainty for agri‐food SMEs in Egypt. The empirical results have important implications for the design and implementation of effective policy interventions to mitigate the direct impacts of the pandemic on agri‐food SMEs and its economy‐wide effects on businesses, employment, and livelihoods.

The results show that the location of the surveyed agri‐food SMEs was a significant determinant of their perception of COVID‐19 risks. Compared to agri‐food SMEs located in OLNDA, those located in NRLDA perceived less risk. This finding is in concert with the findings of a recent OECD (2020a) study showing that the impact of COVID‐19 has a strong territorial dimension that has significant consequences for risk perception and crisis management. An explanation of our finding could be that desert and newly reclaimed areas in Egypt are significantly less populated compared to the Nile Delta provinces, which decreased the possibility of frequent interpersonal contacts and reduced the spread of the pandemic. The slow spread of the virus led to less strict containment measures and mobility restrictions in OLNDA, shortened the duration of temporary business shutdowns, and therefore reduced the negative impacts of the pandemic on agri‐food SMEs' supply chains and market demand. Another explanation could be attributed to the fact that many of the agri‐food SMEs in NRLDA are more export‐oriented, implying that they adopt higher food safety and quality standards, have a greater ability to implement hygiene and health measure to reduce risks in the workplace, and are comparatively well‐equipped and prepared to deal with uncertainty and market shocks.

Perception of COVID‐19 risks was significantly lower for agri‐food SMEs located in OLNDA with longer cash flow coverage periods and higher values of total assets. These findings may be attributed to the inherently risky nature of agribusiness in Egypt, which makes total assets and cash holdings function as a buffer against unexpected shocks. Our findings lend support to Psillaki and Eleftheriou (2015), who show that SMEs generally encounter financial problems even in normal times, including a lack of working capital. Therefore, when they have to shut down temporarily due to extreme events, they have little cash on hand or a suitable cash flow to cover their recurrent expenses, including workers' salaries, interest on loans, and rent. La Rocca et al. (2019) show that having greater cash flows and assets reduces the probability that SMEs will experience financial distress and improves the probability of taking advantage of growth opportunities and achieving success in the long run. Therefore, our findings emphasize the fact that providing government support to agri‐food SMEs during disruptions through low‐interest or interest‐free loans and spatially targeted tax incentives is essential to ease pressure on the SMEs' capital chains and to ensure their survival, business continuity, and recovery.

Our results indicate that having internal guidelines for risk management is associated with higher risk perception among agri‐food SMEs in OLNDA that operate in the domestic market. This finding is in keeping with previous findings by Ates et al. (2013), who showed that SMEs traditionally emphasize short‐term planning, have less codified policies, and formalized structures to deal with unexpected situations, and react to unexpected shocks as they arise, usually with a focus on nothing more than the survival of the business (Hudson‐Smith & Smith, 2007). According to Sullivan‐Taylor and Branicki (2011), their limited managerial resources and financial constraints impede their ability to prepare for and develop risk management strategies. Such attributes and the “just‐in‐time” approach to risk management along with the absence of a proactive and preventative stance to risk management reduce the resilience of agri‐food SMEs to extreme events and leave them with limited opportunities to recover. Therefore, our findings assert that extension services and customized guidance offered by government authorities geared toward supporting enterprise should be focused on targeted awareness‐raising, ex‐ante risk management and prevention, and capacity‐building interventions in accordance with the characteristics of the individual agri‐food SMEs. Such interventions should aim to diminish agri‐food SMEs' risk exposure and enhance their awareness and preparedness arrangements to address risks as well as to anticipate their impacts.

Generally, our findings show that firms that operate both in domestic and export markets for fresh fruit perceive less risk from the COVID‐19 pandemic. As the majority of Egyptian agri‐food export SMEs have their own farms and are more vertically integrated with greater control over the supply chain, these characteristics enable them to strengthen their supply chain, reduce production costs, capture upstream or downstream profits, and mitigate the impacts of supply chain risks. Furthermore, this finding is in line with previous findings by Abu Hatab et al. (2019) indicating that Egyptian agri‐food SMEs that sell in both markets have higher market flexibility and can implement product and market shifts when facing unexpected market shocks. In particular, these enterprises are able to shift their exports from one export market to another or re‐direct their exports to the domestic market as the circumstances in their initial markets change. However, the majority of Egyptian agri‐food SMEs regarded the process of market shifting as difficult or very difficult to manage. Linking our findings with those of Abu Hatab et al. (2019) underscores an important research question which arises with regard to the extent to which agri‐food exporting SMEs, which operate with higher marginal costs, can shift their products to the domestic market during disruptions such as the COVID‐19 pandemic and how this process may affect their competitiveness. This question is left to future research.

Another important finding was that the main destination to which agri‐food SMEs export vegetables represented a determining factor for risk perception. That is, we found that agri‐food SMEs whose main export market is Arab countries (46% of the surveyed enterprises) perceived significantly higher COVID‐19 risks, compared to agri‐food SMEs that export mainly to the EU (33% of the surveyed enterprises) or to other destinations. Although Egyptian agri‐food exporters face stringent nontariff measures in the EU, the existence of trade agreements, such as the EU‐Egypt Association Agreement, seems to facilitate the access of Egyptian agricultural exports to the EU market and protect them from unexpected market shocks (Helmy et al., 2018). Moreover, while most food legislation and import requirements for plant and plant products in the EU are harmonized (Vandercammen, 2019), the main market access barrier to Egyptian agri‐food exports is compliance with these clearly defined and transparently implemented requirements. In contrast, the Arab markets, in particular the Gulf States, are characterized by a high degree of heterogeneity in the restrictiveness of their regulatory systems, trade standards, and import regulations as well as in the presence of procedural obstacles, such as frequently changing laws and short implementation notices (ITC, 2016). During the COVID‐19 pandemic, these characteristics of Arab markets exposed Egyptian agri‐food SMEs to market and price fluctuations, increased their vulnerability to risks, and impeded their ability to adapt production processes quickly and adequately to meet export requirements. Our findings lend support to previous studies (e.g., Dogruel & Tekce, 2010; Siam, 2002) that highlighted the heavy dependency of Egyptian agri‐food exporters on few destination markets, mainly the Arab Gulf States, which leaves them vulnerable during rapid changes and sudden shocks in these markets. Therefore, national policy measures focused on building the resilience of agri‐food SMEs to risks should focus on export diversification, put more effort into regional cooperation, establish the conditions needed for facilitating the access of agri‐food SMEs to new export markets, and create an institutional and regulatory framework that supports agri‐food SMEs' ability to compete in global markets.

While projections suggest that pandemics and natural hazards will occur more often in the future (McKee & Stuckler, 2020), possible avenues for future research should include an examination of the resilience of agri‐food SMEs in developing countries, particularly the conditions and barriers that determine their ability to withstand, adapt, and recover from extreme events.

## Supporting information

Supporting information.

## Data Availability

The data that support the findings of this study are available from the corresponding author upon reasonable request.
