# Supplementary material for: Risk perception and determinants in small‐ and medium‐sized agri‐food enterprises amidst the COVID‐19 pandemic: Evidence from Egypt
Source: Agribusiness (N Y N Y). 2020 Dec 3;37(1):187–212. doi: 10.1002/agr.21676 (PMC7753412; doi:10.1002/agr.21676)
Supplement: Supplementary file 1 — Supporting information. [file AGR-37-187-s001.docx]

**Questionnaire**

**“Risk Management Strategies in Egypt's Small & Medium-Sized Agrifood Firms amidst the COVID-19 outbreak”**

Dear Respondent,

We are conducting a survey to identify the impacts of COVID-19 on the business performance of Egyptian agrifood SMEs and examine the risk management strategies that these firms have adopted and plan to adopt to cope with the pandemic impacts. The research team for this survey is made up of researcher scientists from Egyptian and Swedish universities.

You have received this invitation because your firm is registered as Agrifood SMEs. As a firm manager, we would like to invite you to participate in our study. This survey takes approximately 25 minutes

**Your participation and your answers are very important to provide a good understanding of the situation for Egyptian agrifood SMEs!**

**Please be noted** that the information gathered in this survey will be treated confidentially and will be used for scientific research purposes **ONLY**. The responses provided will not be linked to individual names or addresses. All information that might indirectly identify respondents or firms will be eliminated from the data file before it is made available. Publications based on the data will never contain information that can identify individual respondents.

If you have inquiries, please feel welcome to contact Dr. XXX.

We would like to say thank you in advance for your participation.

Yours faithfully,

| **For Research team use only** | |
| --- | --- |
| **Form Number** |  |
| **Date of the Interview** |  |
| **Interviewer’s Name** |  |

1. **RESPONDENT'S CHARACTERISTICS**
   1. **Gender**

Female ( ) Male ( )

- 1. **Into which age range do you fall? (Please tick one only)**

18-24 ( ) 25-34 ( ) 35-44 ( ) 45-54 ( ) 55-64 ( ) 65-74 ( ) 75+ ( ) Would rather not say ( )

- 1. **Level of education**

No formal schooling ( ) Primary school ( ) Secondary school ( ) Technical qualification ( ) University degree ( ) Higher degree, MSc/Ph.D ( )

- 1. **Position held at the firm**

(a) Chairman of the Board (b) General Manager (c) Director or Deputy General Manager (d) Head of Finance or Accounting (e) Head of Operations (Sales) (f) Technical Director (g) Other: ______

- 1. **How many years have you held your current position within the company?**
  2. **In which governorate is the firm located? (** dropdown menu including a list of 27 Egyptian governorates**)**
  3. **Ownership type**
- Domestic investment
- Foreign Investment
- Joint investment
  1. **Which one of the following best describes the area where the firm is located?(Please tick one only)**
- Rural
- Suburb (peripheral areas)
- Urban
- Industrial zone
- Other (please specify)________________
  1. **Does your firm has any other branches in other locations?**
  2. **If yes, How many and where are they located?**

| **Branches** | **Location** |
| --- | --- |
|  |  |

- 1. **How many years has your firm been working in the agricultural and food business?**
  2. **Size of the Firm: Please indicate the number of people currently employed by your firm.**
  3. **Firm Specialization: What are the major agrifood exports of your firm? And, what is the current percentage (%) of domestic demand and export share of the firm’s product in total output or production?**

| Firm Specialization | | | | |
| --- | --- | --- | --- | --- |
| Domestic | | International % | | |
| % of total sales | Main products | % of total sales | Main products | Main markets |
|  |  |  |  |  |

- 1. **Total assets: Into which of the following categories does the value of total assets of your firm fall?**
- Less than 3000 000 EGP
- 3000 000 to 5000 000 EGP
- 5000 000 to 10 000 000 EGP
- 10 000 000 to 15 000 000 EGP
- 15 000 000 to 20 000 000 EFP
- Over 20 000 000 EGP
  1. **Total annual sales: Into which of the following categories does the value of the annual sales of your firm in 2019 fall?**
- Less than 1000 000 EGP
- 1000 000 to 3000 000 EGP
- 3000 000 to 5 000 000 EGP
- 5 000 000 to 10 000 000 EGP
- Over 10 000 000 EGP
  1. **Please indicate which of the following certification standards your company have at the moment (you can choose as many as you have).**

| Certificate | Company Certified to System |
| --- | --- |
| GLOBAL GAP |  |
| ISO 90001:2008 |  |
| ISO 14001 |  |
| HACCP |  |
| SA 8000 |  |
| BRC |  |
| BASC |  |
| IFS |  |
| SQF2000/2001 |  |
| TESCO |  |
| LEAF |  |
| GMP |  |
| ISO 22000 |  |
| Others |  |
| Others |  |
| Others |  |

- 1. **Does the firm Keep financial records?**
- Yes
- No
  1. **Does the firm has a specialized department/unit to deal with risk management?**
- Yes
- No
  1. **Does the firm has internal "written" guidelines for risk management?**
- Yes
- No

1. **PERCEIVED IMPACTS SO FAR**
   1. **At this point, what are the main business problems your firm is currently facing due to the pandemic?**

(a) Reduction of sales

(b) Inability to deliver existing orders

(c) Increased difficulty of financing

(d) Existing loans cannot be extended

(e) Disruption of logistics

(f) Upstream and downstream chain disruptions

(e) other:

- 1. **At this point, how does the total revenue of your company during April 2020 compare to April 2019?**

(a) Increase by more than 10%

(b) Increase, but less than or equal to 10%

(c) Same as last year

(d) Decrease of less than or equal to 10%

(e) Decrease of more than 10%

(f) Unable to judge

- 1. **At this point, how would you evaluate the cost of your company's raw materials and total operating costs in April 2020 compared to April 2019:**

(a) Increase by more than 10 percent

(b) Increase, but less than or equal to 10 percent

(c) Be the same as last year

(d) Decrease by less than 10 percent

(e) Decrease by more than 10 percent

(f) Unable to judge

- 1. **Because of the epidemic, what is the percentage of your company's employees who are unable to come to work at present?**

(a) 0%

(b) 1-10%

(c) 11-20%

(d) 21-30%

(e) More than 30%

(f) Unable to judge

- 1. **Please choose the most significant financial problems for your company during this period.**

(a) Staff wages and social security charges

(b) Rent

(c) Repayment of loans

(d) Payments of invoices

(e) Other expenses

(f) No specific problem

- 1. **If your company currently considering layoffs, or has already done some because of the epidemic, what percentage of staff are you expecting to (or have already) cut?**

(a) 0%

(b) 1-10%

(c) 11-20%

(d) 21-30%

(e) More than 30%

(f) Unable to judge

- 1. **How long can your company's current cash flow maintain the company's operation?**

(a) Less than 1 month

(b) 1-3 months

(c) 4-5 months

(d) 6 months or more

- 1. **Have you had any consignments rejected during the last three months because of Covid-19?**
  2. **If yes, how frequently are consignments rejected?**

Very frequently

Frequently

Neither frequently nor infrequently

Infrequently

Very infrequently

1. **SOURCES OF RISK TO FIRMS' OPERATIONS**

**Note to the enumerator**: Please provide example to the respondents on how to answer this part of the survey.

COVID-19 represents **unprecedented** situation for most of Egyptian agrifood SMEs. We are now interested in knowing how you expect the coronavirus to impact your business during the period from today until the end of this year.

| Type | **Source of risk** | 1. How severe is the impact of this source on your firm performance? | | | | | 1. What do you think is the likelihood that your firm will be affected by the source of the risk? | | | | | | 1. When in the future do you think you will notice any harmful impact of the source of risk to your firm performance? | | | | |
| --- | --- | --- | --- | --- | --- | --- | --- | --- | --- | --- | --- | --- | --- | --- | --- | --- | --- |
|  |  | Not at all a problem  (0) | Minor problem  (2.5) | Moderate  (5) | Serious problem  (7.5) | Very serious  (10) | None  0 | Very  low  1 | Low  2.5 | Med-ium  5 | High  7.5 | Very  High  10 | Direc-tly  (10) | Within a week  (8) | Within a 3 months  (4) | After longer time  (end of 2020)  (1) | Not at all  (0) |
| Revenue/sales | Decrease in total value of monthly DOMESTIC sales |  | | | | |  | | | | | |  | | | | |
| Revenue/sales | Decrease in total monthly EXPORT sales |  | | | | |  | | | | | |  | | | | |
| Revenue/sales | Loss of revenue from purchasing delays and reduced collection of receivables |  | | | | |  | | | | | |  | | | | |
| Revenue/sales | Reduced purchasing power: consumers may have less money available to purchase food |  | | | | |  | | | | | |  | | | | |
| Supply Chains | Shipment of fresh produce grounded at airports and ports because travel has stopped |  | | | | |  | | | | | |  | | | | |
| Supply Chains | Restrictions on transportation and disruptions in distribution channels to markets |  | | | | |  | | | | | |  | | | | |
| Supply Chains | Market rejections: commodities were refused by the importer/retailer |  | | | | |  | | | | | |  | | | | |
| Supply Chains | Failure to deliver contracted sales to partners due to lockdown |  | | | | |  | | | | | |  | | | | |
| Supply Chains | Delayed port operations and increased pressure on internal transport |  | | | | |  | | | | | |  | | | | |
| Labor | Plummeting Employee Productivity: employees are unable to commute to work (e.g. lockdown) |  | | | | |  | | | | | |  | | | | |
| Labor | loss of skilled labor as experienced employees with valuable information and knowledge and/or contacts leave the firm |  | | | | |  | | | | | |  | | | | |
| Labor | Reduction in the number of working days (open days) |  | | | | |  | | | | | |  | | | | |
| Labor | High rates of work absenteeism |  | | | | |  | | | | | |  | | | | |
| Cost | The cost of commodities sold by the firm increased |  | | | | |  | | | | | |  | | | | |
| Cost | SMEs cannot afford investments for market and technological development |  | | | | |  | | | | | |  | | | | |
| Cost | The cost of inputs and (e.g. labor, fertilizer) increased |  | | | | |  | | | | | |  | | | | |
| Cost | Increased the losses and waste |  | | | | |  | | | | | |  | | | | |
| Cost | Cost of implementing preventative measures at the workplace |  | | | | |  | | | | | |  | | | | |
| Institutions | Reduced capacity of public and private institutions to provide services to SMEs |  | | | | |  | | | | | |  | | | | |
| Institutions | Policy uncertainty with regard to corporate tax |  | | | | |  | | | | | |  | | | | |
| Institutions | Policy uncertainty with regard to cutting employees' numbers and salaries |  | | | | |  | | | | | |  | | | | |
| Institutions | Policy uncertainty with regard to (central) bank's interest rate policies |  | | | | |  | | | | | |  | | | | |
| Institutions | Economic recession or political instability |  | | | | |  | | | | | |  | | | | |
| Institutions | Difficulty to access emergency support introduced by government departments. |  | | | | |  | | | | | |  | | | | |
| Finance | Increased cost of obtaining loans (interest rate) |  | | | | |  | | | | | |  | | | | |
| Finance | Banks and financial sector organizations are unwilling to provide credit to SMEs |  | | | | |  | | | | | |  | | | | |
| Finance | Inability to pay back my loans |  | | | | |  | | | | | |  | | | | |
| Suppliers | Shortage of the quantities of agricultural produce to meet the demands |  | | | | |  | | | | | |  | | | | |
| Suppliers | Reduced quality of agricultural produce to meet the market demands |  | | | | |  | | | | | |  | | | | |

1. **Total risk (Disaggregated)**

Now consider the sources of risk from the previous task. **On the whole what is the relative importance of the risks to your firm from these sources?**

**Distribute marks to each attribute and make sure that the total score sums to 100**

**4.1 Risk for firm's total sales and revenue**

| **Source of risk** | Your score |
| --- | --- |
| Decrease in total value of monthly DOMESTIC sales |  |
| Decrease in total monthly EXPORT sales |  |
| Loss of revenue from purchasing delays and reduced collection of receivables |  |
| Reduced purchasing power: consumers may have less money available to purchase food |  |
| **Total score** | **100** |

**4.2. Risk for firm's supply chain**

| **Source of risk** | Your score |
| --- | --- |
| Shipment of fresh produce grounded at airports and ports because travel has stopped |  |
| Restrictions on transportation and disruptions in distribution channels to markets |  |
| Market rejections: commodities were refused by the importer/retailer |  |
| Failure to deliver contracted sales to partners due to lockdown |  |
| Delayed port operations and increased pressure on internal transport |  |
| **Total score** | **100** |

**4.3 Risk for firm's labor**

| **Source of risk** | Your score |
| --- | --- |
| Plummeting Employee Productivity: employees are unable to commute to work (e.g. lockdown) |  |
| loss of skilled labor as experienced employees leave the firm |  |
| Reduction in the number of working days (open days) |  |
| High rates of work absenteeism |  |
| The cost of commodities sold by the firm increased |  |
| **Total score** | **100** |

**4.4 Risk for firm's cost of production**

| **Source of risk** | Your score |
| --- | --- |
| SMEs cannot afford investments for market and technological development |  |
| The cost of inputs and (e.g. labor, fertilizer) increased |  |
| Increased the losses and waste |  |
| Cost of implementing preventative measures at the workplace |  |
| Inability to pay back firm's loans |  |
| **Total score** | **100** |

**4.5 Policy related risk for firm's operations**

| **Source of risk** | Your score |
| --- | --- |
| Reduced capacity of public and private institutions to provide services to SMEs |  |
| Increase the cost of obtaining loans (interest rate) |  |
| Policy uncertainty with regard to corporate tax |  |
| Unwillingness of banks and financial sector organizations to provide credit to SMEs |  |
| Policy uncertainty with regard to lockdown and preventative measures |  |
| Economic recession or political instability |  |
| Difficulty to access emergency support introduced by the government departments |  |
| **Total score** | **100** |

**Total risk**

| **Source of risk** | Your score |
| --- | --- |
| Risks to sales and revenue (4.2.1) |  |
| Supply chain risks (4.2.2) |  |
| Labor risks (4.2.3) |  |
| Risks to firm's production cost (4.2.4) |  |
| Policy related risks (4.2.5) |  |
| **Total score** | **100** |

1. **RISK/IMPACT MANAGEMENT STRATEGIES**
   1. **The following table includes a list of potential actions/strategies that may be relevant for agrifood SMEs to respond to the impacts of the pandemic. To what extent are these relevant to your firm?**

| **Type** | **Means/Strategy** | **Level of agreement** | | | | |
| --- | --- | --- | --- | --- | --- | --- |
|  |  | Completely disagree | Somewhat  Disagree | Neutral/not relevant | Somewhat agree | Completely agree |
| **Finance/cash shortage** | |  |  |  |  |  |
|  | We will have to seek loans by commercial banks |  |  |  |  |  |
|  | We will have to apply for government assistance programs |  |  |  |  |  |
|  | We will apply for loans by microfinance companies or private individuals |  |  |  |  |  |
|  | We will negotiate with lenders to avoid withdrawing loans |  |  |  |  |  |
|  | We will add new shareholders or increase capital of shareholders |  |  |  |  |  |
|  | We will reduce operating costs (e.g. layoffs and salary reductions) |  |  |  |  |  |
|  | We will buy insurance to cover unexpected events |  |  |  |  |  |
|  | No cash flow shortfalls problem, so no action |  |  |  |  |  |
| **Fulfilling contracts** | |  |  |  |  |  |
|  | We will negotiate our contracts to settle them by mutual agreement |  |  |  |  |  |
|  | We will seek legal settlement for our unfulfilled contracts |  |  |  |  |  |
|  | We will have to make payments of liquidated losses |  |  |  |  |  |
|  | We will procure through loyal suppliers |  |  |  |  |  |
|  | We will reach out to vendors and ask for payment term extensions |  |  |  |  |  |
|  | No contractual performance issues, so no action |  |  |  |  |  |
| **Supply chain** | |  |  |  |  |  |
|  | We will shut down operations or segments of operations |  |  |  |  |  |
|  | We will change the operation strategies (production, marketing) |  |  |  |  |  |
|  | We will procure inputs in local markets |  |  |  |  |  |
|  | We will network with other firms to help prevent risky transactions |  |  |  |  |  |
|  | We will outsource orders |  |  |  |  |  |
|  | We will increase the prices |  |  |  |  |  |
|  | We will seek new procurement channels |  |  |  |  |  |
|  | We will have to delay products'' delivery |  |  |  |  |  |
|  | We will innovate new products and services |  |  |  |  |  |
|  | We will introduce new customer delivery options |  |  |  |  |  |
|  | We will focus on maintaining and improving quality of goods sold |  |  |  |  |  |
|  | We will shift our products towards other markets rather than traditional markets |  |  |  |  |  |
|  | We will shift from selling our traditional products to products |  |  |  |  |  |
|  | No supply chain issues, so no action |  |  |  |  |  |
| **Labor/work environment** | |  |  |  |  |  |
|  | We will reduce payroll costs by cutting down the number of employees |  |  |  |  |  |
|  | We will reduce workweek (working days) |  |  |  |  |  |
|  | We will apply salary cutbacks for personnel |  |  |  |  |  |
|  | We will promote work-from-home options |  |  |  |  |  |
|  | We will implement preventive measures and maintain a safe workplace |  |  |  |  |  |
|  | We will increase wages to prevent employees leaving the firm |  |  |  |  |  |
|  | No labor issues, so no action |  |  |  |  |  |
| **Other strategies** | |  |  |  |  |  |
|  |  |  |  |  |  |  |
|  |  |  |  |  |  |  |

- 1. **Which types of competence do you have in relation to coping with the risk dimensions of the pandemic**

| **Items** | **Completely disagree** | **Somewhat**  **Disagree** | **Neutral/not relevant** | **Somewhat agree** | **Completely agree** |
| --- | --- | --- | --- | --- | --- |
| Management and control systems |  |  |  |  |  |
| Safety and quality of raw agricultural products |  |  |  |  |  |
| Skilled labor |  |  |  |  |  |
| Adequate technology |  |  |  |  |  |
| Capital and financial resources |  |  |  |  |  |
| Knowledge and R&D |  |  |  |  |  |
| Price competitiveness |  |  |  |  |  |
| Brand recognition |  |  |  |  |  |
|  |  |  |  |  |  |
|  |  |  |  |  |  |
|  |  |  |  |  |  |
|  |  |  |  |  |  |
|  |  |  |  |  |  |

1. **EMERGING OPPORTUNITIES (BENEFITS)**
   1. **To what extent would you agree with the following statements regarding the positive changes and opportunities that the pandemic may have offered to your firm's business?**

| **Items** | **Completely disagree** | **Somewhat**  **Disagree** | **Neutral/not relevant** | **Somewhat agree** | **Completely agree** |
| --- | --- | --- | --- | --- | --- |
| Improved our access to government assistance programs |  |  |  |  |  |
| Facilitated our access to bank loans and financial services and |  |  |  |  |  |
| Increased market prices for sold commodities |  |  |  |  |  |
| Improved our efficiency in terms of resource use (e.g. labor) |  |  |  |  |  |
| Offered new opportunities in local markets |  |  |  |  |  |
| Offered new opportunities within regional markets in Egypt |  |  |  |  |  |
| Offered new opportunities in the international market |  |  |  |  |  |
| Enabled us to introduce new products |  |  |  |  |  |
| Allowed us to adopt new operational strategies to diversify products and penetrate markets |  |  |  |  |  |
| Allowed us to adopt new operational strategies to innovate new services |  |  |  |  |  |
| Allowed us to introduce new customer services and delivery options |  |  |  |  |  |
| Enhanced our preparedness for future risks |  |  |  |  |  |
| Improved our ability to realize return on investment |  |  |  |  |  |

1. **LONG-RUN IMPACTS**
   1. **How would you estimate the cost of adaptation to the risks and challenges posed by the pandemic, as a percentage in your total earnings in 2020?**

Less than 5 %

5-10%

10-20%

20-30%

30-40%

40-50%

50-60%

More than 60%

- 1. **If your company is an export-oriented enterprise, how do you expect the epidemic to affect your company's export volume?**

(a) Increase by more than 10%

(b) Increase, but less than or equal to 10%

(c) Same as last year

(d) Decrease of less than or equal to 10%

(e) Decrease of more than 10%

(f) Unable to judge

- 1. **What is the expected time for the firm's business recovery?**

(a) 1 month to 3 months

(b) 3 -6 months

(c) 6-9 month

(d) 9-12 months

(e) more than a year

(f) Inability to judge

- 1. **How adequate do you consider your own capacity for adapt to the challenges posed by the pandemic?**

Totally adequate

Somewhat adequate

Neither adequate nor inadequate

Somewhat inadequate

Totally inadequate

- 1. **How does your capacity to adapt to the challenges posed by the pandemic compared to that of other Egyptian agrifood SMEs?**

Much higher

Higher

Same lower

Much Lower

1. **FUTURE FOLLOW-UP**
   1. **What is the full name of your company? (Optional)**
   2. **We would greatly appreciate your participation in a follow up survey in a few months. If you’d like to participate, please leave your contact details (optional, mobile, email or landline number).**

***Thanks for your Time and Cooperation***
